# Phylogeography of Puumala orthohantavirus in Europe

**DOI:** 10.3390/v11080679

**Published:** 2019-07-24

**Authors:** Guillaume Castel, François Chevenet, Maria Razzauti, Séverine Murri, Philippe Marianneau, Jean-François Cosson, Noël Tordo, Alexander Plyusnin

**Affiliations:** 1 CBGP, INRA, CIRAD, IRD, Montpellier SupAgro, Université Montpellier, 34000 Montpellier, France; 2 MIVEGEC, Université de Montpellier, CNRS, IRD, 34394 Montpellier, France; 3 LIRMM, Université de Montpellier, CNRS, 34095 Montpellier, France; 4 ANSES, Virology Unit, 69007 Lyon, France; 5 BIPAR-INRA Biologie Moléculaire et Immunologie Parasitaires et Fongiques, ENVA Maisons Alfort, 7 Avenue du Général de Gaulle, 94704 Maisons-Alfort CEDEX, France; 6 Institut Pasteur, Antiviral Strategies Unit, Department of Virology, Paris, France & Institut Pasteur de Guinée, BP 4416 Conakry, Guinea; 7 Department of Virology, University of Helsinki, 00014 Helsinki, Finland

**Keywords:** puumala orthohantavirus, phylogeography, co-evolution, bank vole (myodes glareolus)

## Abstract

Puumala virus is an RNA virus hosted by the bank vole *(Myodes glareolus)* and is today present in most European countries. Whilst it is generally accepted that hantaviruses have been tightly co-evolving with their hosts, Puumala virus (PUUV) evolutionary history is still controversial and so far has not been studied at the whole European level. This study attempts to reconstruct the phylogeographical spread of modern PUUV throughout Europe during the last postglacial period in the light of an upgraded dataset of complete PUUV small (S) segment sequences and by using most recent computational approaches. Taking advantage of the knowledge on the past migrations of its host, we identified at least three potential independent dispersal routes of PUUV during postglacial recolonization of Europe by the bank vole. From the Alpe-Adrian region (Balkan, Austria, and Hungary) to Western European countries (Germany, France, Belgium, and Netherland), and South Scandinavia. From the vicinity of Carpathian Mountains to the Baltic countries and to Poland, Russia, and Finland. The dissemination towards Denmark and North Scandinavia is more hypothetical and probably involved several independent streams from south and north Fennoscandia.

## 1. Introduction

Puumala virus (PUUV) belongs to the order *Bunyavirales*, family *Hantaviridae*, genus *Orthohantavirus* [1]. It was first isolated in 1979 from bank voles (*Myodes glareolus*) in Puumala (Finland) [2], as a virus related to but distinct from the prototype orthohantavirus, Hantaan virus (HTNV), discovered in Korea [3]. Hantavirus genome comprises three negative-stranded RNA segments: The large (L) segment encoding the viral RNA-dependent RNA polymerase, the medium (M) segment encoding the envelope glycoproteins Gn and Gc precursor (GPC), the small (S) segment encoding the viral nucleocapsid protein [4], and, for some hantaviruses, also the nonstructural protein (NSs) [5]. PUUV is the causative agent of nephropathia epidemica (NE), a mild form of an hemorrhagic fever with renal syndrome (HFRS) [6] transmitted by infected bank voles.

The bank vole is widespread from the Mediterranean countries to Scandinavia, and from Great Britain up through Russia (Ural, Siberian Taiga—Omsk) [7]. Its population dynamics over the past two decades [8] has led to a geographic expansion provoking an increased number of NE cases [9,10]. Today PUUV is found in most European countries [11] with about 10,000 cases of NE reported annually, mostly in Fennoscandia (Scandinavian Peninsula, Finland, Karelia, and the Kola Peninsula). However, NE is probably strongly underdiagnosed in many countries due to suboptimal surveillance and underreporting of benign cases [6,12].

To date, eight PUUV lineages have been described in Eurasia [13,14]: The Central European (CE) lineage is spread in France, Belgium, Germany, Netherland, and Slovakia; the Alpe-Adrian (ALAD) lineage covers Austria, Slovenia, Croatia, and Hungary; the Latvian (LAT) lineage is observed in Latvia, Lithuania, and Poland; the Danish (DAN) lineage in the island of Fyn (the third-largest island of Denmark belonging to the region of Southern Denmark); the South-Scandinavian (S-SCA) lineage from Norway to central and southern Sweden; the North-Scandinavian (N-SCA) lineage from northern Sweden to northwestern Finland; the Finnish (FIN) lineage covering Finland, Russian Karelia, and western Siberia (Omsk region); and the Russian (RUS) lineage including isolates from pre-Ural Russia and from Baltic countries (Estonia and Latvia). These PUUV lineages show strong geographical clustering [13,15,16,17,18]. Based on the S segment nucleotide sequence, intra- and inter-lineages diversity reaches 0.3–9.0% and 15–27%, respectively (see [13]).

It is generally accepted that hantaviruses have tightly co-evolved (co-diversified) with their mammal hosts explaining the parallelism between orthohantavirus and rodent phylogenies [13,19,20,21]. However, other authors propose that coevolution resulted from the recent colonization of rodents by hantaviruses, a phenomenon referred to as phylogenetic tracking [22,23]. 

PUUV genetic diversity can be driven by both genetic drift, i.e. the continuous accumulation of nucleotide substitutions and/or small insertions/deletions [24,25,26] and genetic shift, i.e., the reassortment of genome segments of isolates of the same or different species infecting the same host cell [25,26,27,28]. Genetic shift includes also recombination [4,13,29]. Indeed, negative (purifying) selection remains the principal driver of PUUV evolution [13,30,31,32] whereas recombinant and reassortant virus variants did not show a competitive advantage to their parental variants and rapidly disappeared [14,26,29].

PUUV evolutionary history has been originally studied at the regional level and/or from relatively small datasets of complete S segments [13,17,32]. It was suggested that an early split had resulted in the current diversification of PUUV lineages [13]. However, recent phylogeographic methods [33,34] applied to the whole Orthohantavirus genus concluded that geography might also impact on PUUV evolution. Using the Bayesian method, Souza et al. (2014) suggested that orthohantaviruses harbored by *Murinae* and *Arvicolinae* subfamilies had been originated in Asia and then spread toward Siberia, Europe, Africa, and North America [35]. Phylogeographic theories cannot be firmly established on a small sample of sequences [36]. Recent Bayesian and maximum likelihood (ML) inference methods for phylogeographic analyses applied to a comprehensive sampling of viruses through time offer promising tools to explore genotypic and phenotypic virus evolutionary history and to predict virus emergence and spread [37]. 

The present study aims to investigate the dynamics and the evolutionary history of PUUV at the European level in the light of a lately upgraded dataset of complete genomic S segments of PUUV in GenBank. Here, two recent inference methods used for ancestral area reconstruction are compared to investigate the consistency of what is currently known on the PUUV evolutionary history and its host geographic dispersal.

## 2. Materials and Methods

### 2.1. Dataset Acquisition

The dataset is constituted by complete S segment (Open Reading Frame of 1299 nt encoding the viral nucleocapsid protein) of PUUV and PUUV-like sequences of isolates of known location available in Genbank. Some sequences were intentionally not included into the dataset to maintain a balanced number of PUUV sequences among the eight recognized genetic lineages (CE, ALAD, N-SCA, S-SCA, RUS, LAT, DAN, and FIN) and/or sampling locations. Thirteen new French isolates (CE lineage) were sequenced as described in [38] and deposited in GenBank (accession numbers MK946422–MK946434). The 152 selected isolates, their genetic lineages, and their assigned geographic origin are shown in Table S1.

Note that the name of the genetic lineages to which the isolates belong does not always match the original sampling location (Table S1): For example some isolates from Baltic countries belong to the RUS PUUV lineage. As in the phylogeographical analyses, each PUUV isolate is precisely assigned to its geographical origin (sampling location), it can thus differ from the name of its genetic lineage: Alpe-Adria, a bioregion in Central Europe, for isolates from Austria, Hungary, and the Balkans; Baltic (Balt) for isolates from Lithuania, Estonia, and Latvia; Poland (Pol); Belgium (Bel); Denmark (Den); France (Fra); Germany (Ger); Netherland (Net); Nord-Scandinavia (N-Sca) for isolates from the north of Norway and Sweden; South Scandinavia (S-Sca) for isolates from the south of Norway and Sweden; and Finland (Fin) and Russia (Rus). PUUV-like isolates from Asia (Asia) correspond to the Asian Hokkaido (HOKV), Fusong (FUSV) and Muju (MUJV) viruses [39,40,41].

### 2.2. Phylogenetic Analyses

The Clustal Omega alignment program implemented in SEAVIEW v4.4.2 [42] was used for multiple sequence alignments. The ML approach implemented in PhyML v3.0 [43] was used for phylogenetic reconstruction with a statistical approximate likelihood ratio test (aLRT) of branch support. The optimal substitution model was identified as the general time reversible (GTR) +G +I model using SMS software [44] available online on the ATGC web platform [45]. The transition/transversion ratio was fixed to four and nucleotide frequencies were optimized from the data set. Rate heterogeneity was applied using discrete gamma distribution with four rate categories, and with an estimated gamma shape parameter alpha of 0.707 and an estimated proportion of invariable site of 0.476. Phylogenetics trees were visualized using FigTree v1.4.2 [46].

### 2.3. Phylogeographic Analyses

From different methods of ancestral character states reconstruction, we selected Bayesian- and maximum likelihood-based probabilistic methods that have some optimality guaranty [47,48] and compared their output in order to highlight and discuss consonant and incongruent results.

#### 2.3.1. Bayesian Method

Bayesian analyses were performed using the Metropolis-coupled Markov chain Monte Carlo (MCMC) method in BEAST package v1.10.4 [49]. BEAUTi v1.10.4 [49] was used to define parameter settings and to generate BEAST .xml input files. The dataset was analyzed under the GTR +G +I model and a lognormal relaxed clock (allowing branch lengths to vary according to an uncorrelated lognormal distribution). The non-parametric and very flexible coalescent Bayesian skyline tree prior [50] allowing the population size to vary stochastically through time [51,52] together with a symmetric diffusion model in which the transition rates between locations are reversible were used for the reconstruction. The spatial information of the PUUV genetic variants was hence used to infer the geographic patterns of PUUV dispersal by fitting a standard continuous-time Markov chain (CTMC) model. We used Bayesian stochastic search variable selection (BSSVS) procedure [53] that allows for assessing the significance of each migration route through a Bayes factor (BF) test. No outgroup taxa were necessary for this analysis; the sampling dates of the sequenced isolates were used to estimate the evolutionary rate and the ancestral time at the internal nodes. A random tree was used as the starting tree. Since there is no adequate information, the prior “location clock rate” was set as default (CTMC Rate Reference) [54]. All other priors were left to default settings. We performed five independent runs of 50 million generations with parameters sampled every 5000 generations in order to increase the ESS values. Parameters and convergence were evaluated using Tracer v1.7.1 [55] and summary maximum clade credibility (MCC) trees were generated using TREEANNOTATOR v1.10.4 [49], after discarding the first 10% of the trees as burn-in as determined graphically using Tracer v1.7.1 and combination of the five runs by LogCombiner v1.10.4 [49].

#### 2.3.2. Maximum Likelihood Methods

To perform the ancestral character state reconstructions by ML we used two recently published programs: PastView [56,57] and PastML [58,59]. Both methods are based on a F81-like marginal posteriors inference [60] with an optimized scaling factor. Analyses were performed on the rooted phylogenetic tree previously computed by PhyML, with annotated tips (geographical origin of each isolate). The specificity of the PastML method is the use of a decision-theory concept and a Brier criterion to predict a unique state if the node is associated with low uncertainty, or several state if this uncertainty is high [59]. Reconstructions were performed as recommended by the authors (marginal posterior probabilities approximation (MPPA) under a F81-like model). An estimate-from-tips (EFT) model in which the equilibrium frequencies are calculated based on the tip state proportions was also tested.

### 2.4. Visualization and Analyses of the PUUV Dispersion Pathways

Beast results were visualized online using the scenario panel function of Aquapony software [61] available online on the ATGC web platform [62]. PastML results were visualized directly online as zoomable html maps on the PastML webserver [58]. Pastview results were visualized and compared with results obtained by the different methods to find common patterns in multiple evolutionary scenarios with the dedicated functions of the software. We use SPREAD3 to calculate BFs and posterior probabilities (PPs) from BSSVS analysis results, in order to test for statistically significant epidemiological links between discrete locations [63].

## 3. Results

### 3.1. Phylogenetic Analyses of PUUV S Segment Sequence Dataset

**Figure 1:**
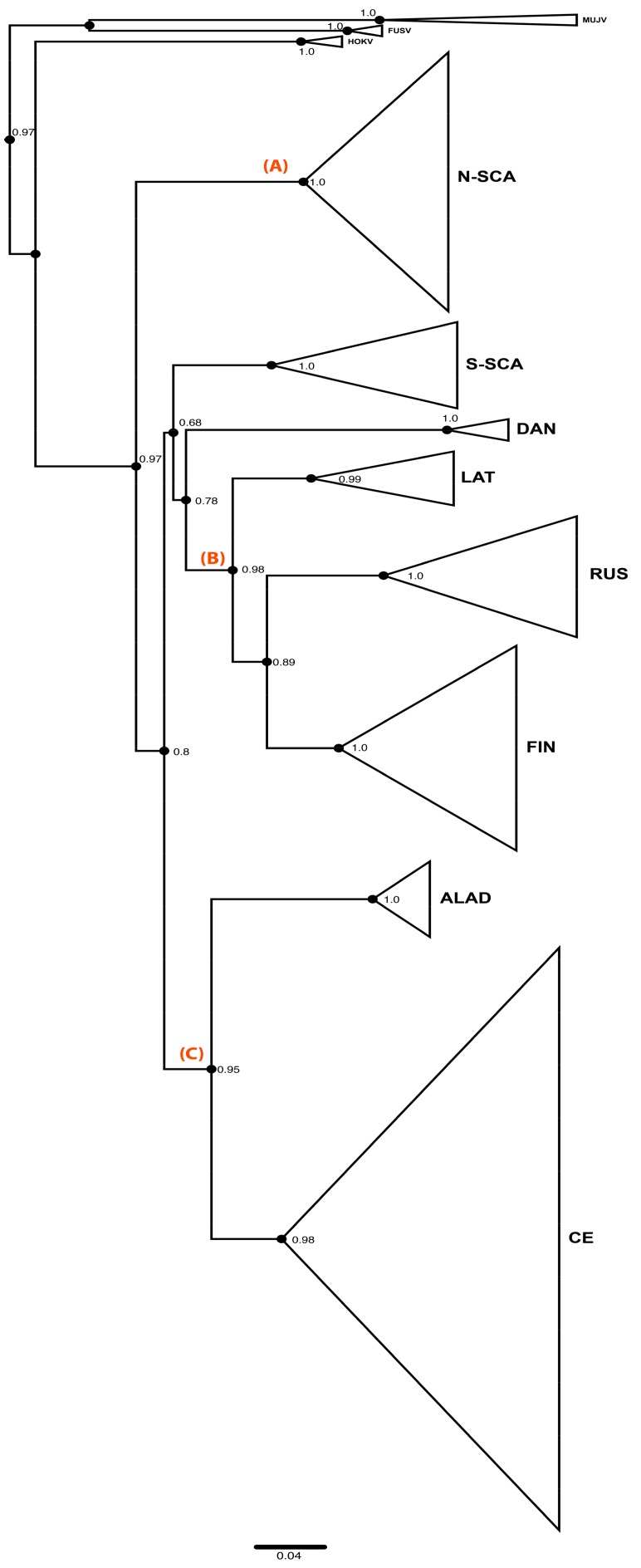
Puumala virus (PUUV) Phylogenetic tree constructed from the complete coding sequence of the small (S) segment by maximum likelihood (ML) method implemented in PhyML 3.0 under the general time reversible (GTR) +G+I substitution model. Branches of the tree clustered within a same PUUV lineage are collapsed to make overall tree visually clear. Eight known PUUV lineages are indicated. The list of sequences belonging to each lineage is indicated in Table S1 together with their geographic origin. Nodes representing most recent common ancestor of North-Scandinavian (N-SCA) lineage, Finnish (FIN), Russian (RUS) and Latvian (LAT) lineages, and Alpe-Adrian (ALAD) and Central European (CE) lineages are indicated, respectively, by the letters A, B, and C.

Figure 1 shows the phylogenetic tree distinguishing the eight previously described genetic lineages of PUUV [13,14]: N-SCA, S-SCA, DAN, LAT, RUS, FIN, ALAD, and CE. PUUV-like HOKV, MUJV, and FUSV viruses (Asian variants) are clearly separated and are basal to the eight PUUV lineages in the tree (Figure 1).

The PUUV phylogeny was previously described as “star-like” (see [13]) suggesting an early split and a radiated spread of all genetic lineages from a single spot. Indeed, the eight PUUV lineages form several groups [13,64]. The ALAD and the CE PUUV lineages, including isolates from the Alpe-Adria region and from Western Europe countries (France, Germany, Belgium, and Netherland), respectively, share a common ancestor (node C) as well as the FIN, the RUS, and the LAT PUUV lineages that are somewhat closer to each other (node B). RUS lineage includes isolates from Estonia and western Latvia and from Russia (Samara, Udmurtia, and Bashkortostan). The FIN PUUV lineage gathers sequences from Finland, from the Russian Karelia, but also from the Russian Omsk region (West Siberia). The DAN and S-SCA PUUV lineages, although they do not cluster together with the FIN, the RUS, and the LAT PUUV lineages, are somehow related to them (but with low support). N-SCA PUUV lineage is more isolated from the others (node A), as already pointed out [26].

### 3.2. Phylogeographic Reconstructions

**Figure 2:**
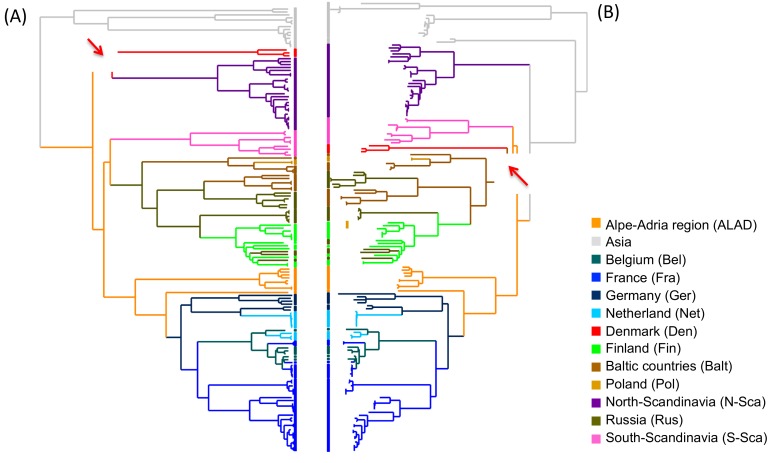

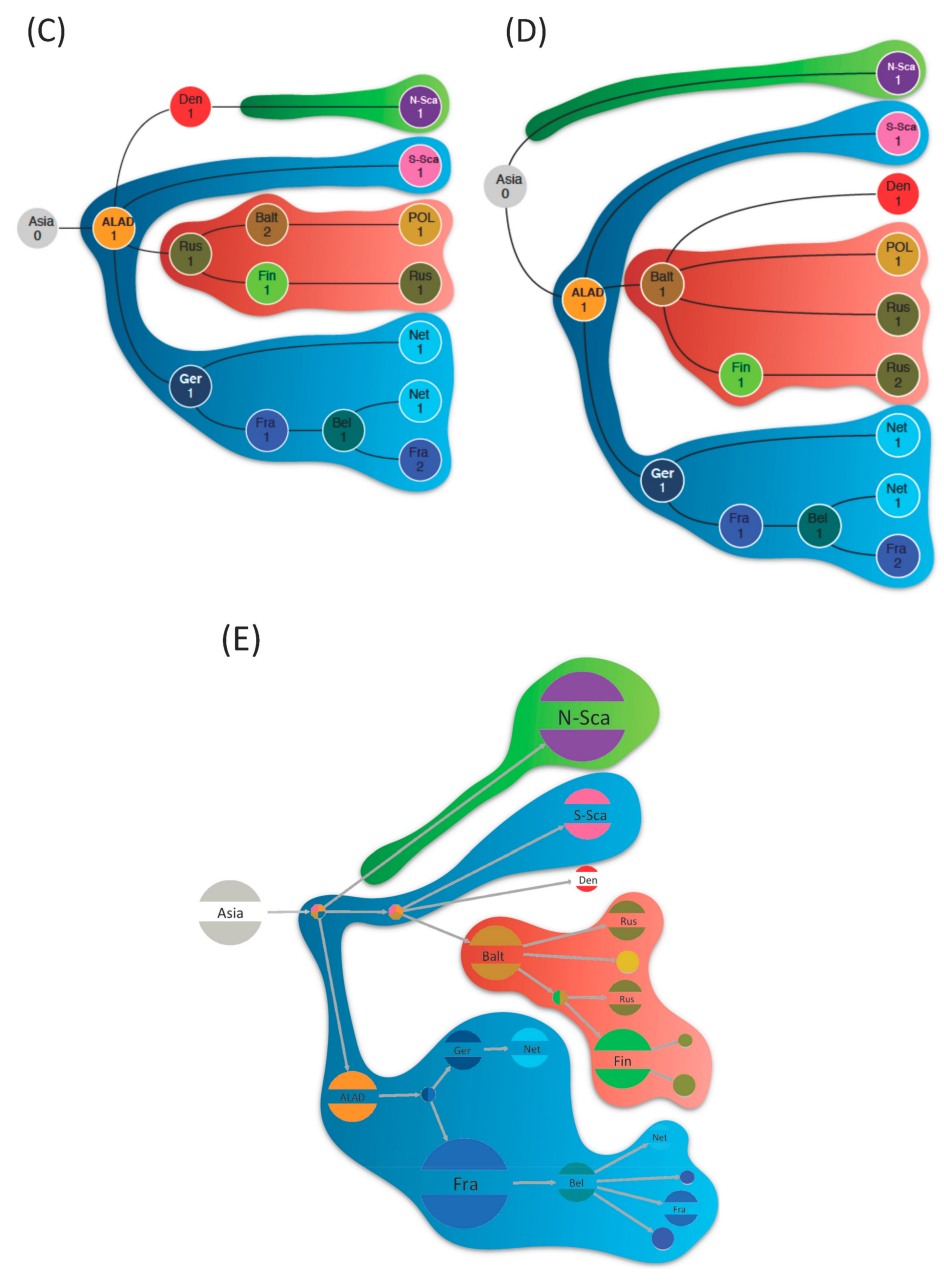
Ancestral geographical reconstructions based on phylogenetic trees obtained by the Bayesian method (Beast program) (**A**) and ML methods, F81 algorithms, (PastView program) (**B**) from the complete coding sequence of the S segment. The red arrows point the nodes in disagreement between Bayesian and ML methods. Tree-like representations of transitions were computed with BEAST (**C**), PastView (**D**), and PastML (**E**) programs. For (**C**) and (**D**), transition maps are summarized with PastView program and numbers indicate the counts of identical transitions having the same ancestor. For (**E**), transition map is done by PastML program and circle diameters are proportional to the number of tips of the initial tree contained in each cluster. Consensus transitions between the three programs are highlighted in blue, red, and green.

In phylogeography, the root of the tree designates the origin of diffusion of the sequence panel available. The two reconstruction methods used in this manuscript, i.e. the Bayesian (Figure 2A) and the ML (Figure 2B), found PUUV Asian variants basal to their European relatives and support the previously suggested hypothesis of an Asian origin of the European lineages [33]. The two phylogeographical methods were also globally consistent and only disagreed concerning the DAN PUUV lineage, which shows a slightly different topology (red branches) in the tree. In general, ancestral reconstructions by ML algorithms, using the PastML program gave the same results whatever the algorithm (F81 or EFT) used. Likewise, the predicted diffusion pathways were similar using both PastView and PastML programs. However, PastML was more cautious in the determination of some ancestors, emphasizing that these steps of the global spread need to be carefully considered. 

We found evidence for three main dispersal routes for PUUV in Europe, whatever the reconstruction method used (Figure 2C–E). The Alpe-Adrian region seems to have played a very central role in this dispersion. From Alpe-Adrian countries, the first route (outlined in blue in Figure 2C–E) generated the S-SCA PUUV lineage on the one hand, strongly supported by all methods (Table S2). On the other hand, it spread across Western Europe through Germany then France and finally the Ardennes forest region bordering Belgium, where sequences are clustered and share the specific Q64 aa signature [13,65,66]. From there, PUUV would have entered in the Netherlands while another lineage would have come directly from Germany. This dissemination pathway shows strong correlation whatever the used method/algorithm (Figure 2C–E) and is supported by good BF and PP (Table S2). 

The second dispersal route (in red in Figure 2C–E) also originated from Alpe-Adrian countries and disseminated into Central-North/Eastern Europe (Finland, Baltic countries, Poland, and Russia). Whilst all methods identify this route, they diverge to identify the starting point of the dissemination. ML methods point out the Baltic countries as a plausible origin of PUUV that would have then spread independently to Poland, Finland, and Russian regions. Russian isolates constitute two clusters, indicating at least two separate introductions. Contrariwise, Bayesian ancestral reconstruction supports a more eastern origin from Russia as the gateway to northern Europe, then, the virus would have spread independently to Baltic countries then Poland, and to Finland/Russian Karelia region. Examination of the probability distribution associated with these transitions shows that the Baltic and Russian origins of this dispersal route have almost equal probabilities (Figure S1A,B). This disagreement already pointed out by Sironen et al. [13], could be due to the potential hybrid origin of the LAT PUUV lineage [14] possibly involving reassortment or recombinant evolutionary processes. The transitions between the locations constituting this dispersal route are well supported by BF and PP (Table S2).

The third diffusion route of PUUV (in green in Figure 2C–E) concerns the N-SCA and DAN PUUV lineages with different scenarios having almost equal probability. As mentioned earlier, the ML and Bayesian methods are inferring different tree topology resulting in different ancestral reconstructions: PastView is in favor of an ancestral introduction of PUUV in North-Scandinavia and a more recent introduction in Denmark from the Baltic region (with no clear decision from PastML). BEAST identifies the most likely scenario as a direct dispersal from ALAD countries to Denmark followed by dissemination to North-Scandinavia. However, a more ancestral and independent introduction of PUUV in North-Scandinavia (as suggested by ML algorithm) is retrieved within the alternative scenario of BEAST results with very close probability (Figure S1C).

## 4. Discussion

Time scale of the orthohantavirus diversification remains controversial today: Assuming ancient adaptation and codivergence with its host *Myodes glareolus*, PUUV evolution rate was initially estimated to be approximately 10^−7^ nucleotide substitutions/site/year [13,30]. Using recent Bayesian coalescent method, PUUV evolution rate was reassessed to about 10^−4^ substitutions/site/year [22,23,35]. However, this method calculates mutation rates from the tips of the tree, what is suitable for short-term evolution but less accurate to estimate ancient divergence events [21,31,67]. Moreover, for hantavirus genes that are evolving under strong purifying selection [13,31,68], the use of classical models [69] can lead to severe underestimation of divergences for viral ancestors [69,70] since deleterious mutations, naturally purged by purifying selection, artificially inflate the evolution rate estimates [23]. Indeed, hantaviruses causing persistent infections tend to codiverge with their host species over extended periods of time (millions of years) and evolve slowly for RNA viruses [71]. The age of hantaviruses may be ten to hundred times older than estimated by classical non-spatial methods [34] and molecular clock-based estimates showing a very recent inter-specific hantavirus evolution are likely to be erroneous [72]. More probably, the phylogeographic pattern of PUUV reflects that of its natural host, supporting the scenario of a virus/host co-evolution/diversification [73,74,75]. In the present study we used phylogeographic approaches to reconstruct the PUUV evolutionary history in the light of the knowledge on the mass migrations of its natural bank vole host throughout Europe during the last postglacial period (1–25 thousand years ago (TYA)).

Our analysis support previous phylogenetic studies suggesting an Asian origin of the current European PUUV lineages [33,35]. The PUUV dissemination from Asia to Europe likely occurred millions of years ago, long before the last glaciation. This climatic event was a new founding event that has left signatures in PUUV lineages that survived with their rodent host population in glacial refugia [76,77]. Isolation in refugia led to the differentiation of several bank vole phylogroups [78] that further recolonized the European landscape (10–25 TYA) [79]. Several possible refugia sites have been identified in the Iberian Peninsula, Alpennines, Balkans, Carpathians, Ukraine, and the Ural Mountains [79,80,81,82]. Bank vole mitochondrial DNA (mtDNA) revealed that eight bank vole phylogroups currently exist referred to as Basque, Spanish, Italian, Balkan, Carpathian, Western, Eastern, and Ural [81,82,83,84] but the postglacial recolonization of Central and Northern Europe has been rather performed by Carpathian, Western, Eastern, and Ural phylogroups [85].

Western bank vole phylogroup arose via expansion from a Central European refugia [83], likely located in the vicinity of the Alps (in the contemporary Alpe-Adrian region) [81] up to France and South Scandinavia through a land-bridge connecting Denmark and southern Sweden at the end of glaciation [86]. Bank voles from the Ural phylogroup migrated from Russian Ural Mountains to the North of Fennoscandia. The origin of bank voles belonging to the Eastern and Carpathian phylogroups remains more controversial. Authors suggest refugia from North-Mediterranean areas in the vicinity of Carpathian Mountains for both phylogroups [27,81]; others are in favor of more eastern refugia close to the contemporary Ukraine for the Eastern phylogroup [83,87]. Then bank voles migrated northward, up to Denmark, Finland, and Russia [14,27,76,83,88,89]. Obviously, migrations of these different phylogroups led to several contact zones where PUUV may circulate today; one between the Ural and Western phylogroups is located in northern Sweden and Norway [76,89,90], another between the Eastern and Ural phylogroups crosses north-central Finland [76,91], and a third one between the Eastern and Carpathian phylogroups in Latvia [14,82,92] (See for a synthesis Figure 1 of [93]).

**Figure 3:**
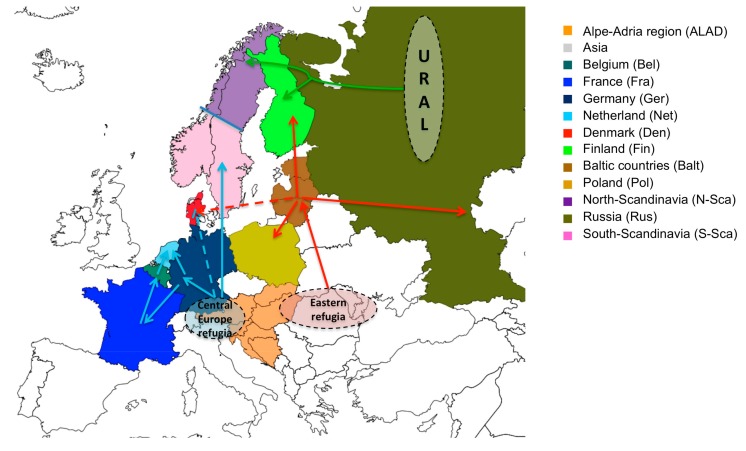
Global view of the waves of post-glaciation dispersal of PUUV in Europe. The three main identified dispersion routes are represented by blue, red and green arrow. Potential routes of PUUV to Denmark are represented by dashed arrows. Dashed circles represent assumed bank vole glacial refugia from which PUUV spread into Europe.

Our phylogeographical results predict at least three main post-glaciation dispersal routes of the PUUV in Europe (Figure 3). 

All used methods agreed that the dispersion of the virus currently present in Western European countries (Germany, France, Belgium, and Netherland) and in South Scandinavia started from the Alpe-Adrian region (Balkan, Austria, and Hungary) (Figure 3, blue route). This confirms the close relationship already observed between PUUV ALAD and CE lineages both carried out by bank voles from the western phylogroup that survived in the Central European refugia during last glaciation [64]. The second dispersal route concerns the PUUV lineages present today in Baltic countries, Poland, Russian, and Finland (Figure 3, red route). Here again, all the methods are in agreement and only slightly differ concerning the exact route to the different countries. They match with dissemination by bank vole of the eastern phylogroup that expanded from an “Eastern” refugia located somewhere between the vicinity of the Carpathian mountains and the contemporary Ukraine towards Baltic sea coast up to central Finland, Denmark, and western Russia (see [27,76]). The PUUV dissemination towards Denmark and North Scandinavia represents the third identified route (Figure 3, green route) for which the used algorithms showed certain inconsistency. PUUV seems to have been transported there by the Ural bank vole phylogroup inhabiting today northern Sweden and northern Finland [27]. 

Interestingly, the S segment of the DAN PUUV lineage are clearly different from those of S-SCA PUUV lineage despite their geographical proximity [17] as is also the case for N-SCA and S-SCA PUUV lineages, known to coexist in Sweden (including in the contact zone) [94]. Our results indicating several independent introductions of PUUV in Fennoscandia are in agreement with these previous studies [89]. One migration came from the south and colonized southern Scandinavia (blue route). Other migrants arrived from the southeast (red route) and northeast (green route) and colonized central Finland and northern Fennoscandia, respectively [76], each phylogroup of bank voles carrying its own genetic variant of PUUV [17]. 

PUUV lineages may have opportunistically cross borders and be associated with different bank vole’s phylogroups during post-glacial recolonization [14,27,76,95]. For example, FIN and N-SCA PUUV lineages co-circulate in the Ural bank vole phylogroup at Pallasjärvi in northern Finland [27], and the RUS and the LAT PUUV lineages have been found in the Carpathian bank vole phylogroup in Jelgava (western Latvia) [14,82]. This spreading of PUUV lineages through bank vole phylogroups in their contact zones suggests that the bottleneck for co-evolution would have been less linked to bank vole/PUUV compatibility than to opportunistic geographical constraints during recolonization [82].

Previous studies suggested that the current PUUV lineages could have resulted from a host-switch of HOKV from *M. rufocanus* to *M. glareolus* around 15 TYA before being dispersed by different lineages of the *M. glareolus* during the post-glacial recolonization [76]. Thus, PUUV would be a “young” virus in bank vole rather than the result of a long co-evolutionary process. However, this hypothesis is conflicting with the high genetic variation observed between the different PUUV lineages regarding the strong purifying selection process to which hantaviruses are exposed. Moreover, as PUUV infections is chronic, non-pathogenic and mainly asymptomatic in *M. glareolus* [96], it is probable that a prolonged coevolution between the virus and its reservoir host has led to the selection of variants having few detrimental effects on the health of the host to ensure propagation [20,97,98]. 

It is sticking, that in large territories of Europe the bank vole populations are apparently free from PUUV, what is theoretically incompatible with the theory of a parallel dispersal of PUUV [11]. For instance, the territory of southern Sweden below the *limes norrlandicus* (the climatic and biological borderline that separates the north and south of Scandinavia), is considered populated by PUUV-free bank voles that are however competent to replicate the virus in laboratory conditions [99]. Several hypotheses have been proposed to explain how the virus vanished from the local rodent population: Insufficient host density or fragmented populations, presence of high levels of maternal Ab to prevent/reduce PUUV persistence locally [11,100,101], host population immunogenetics [11,102], and climatic and/or environmental impacting the virus persistence in the environment [11,103]. 

## 5. Conclusions

In summary, based on the large collection of complete S-segments available in Genbank, our results support that the postglacial dispersal of PUUV into Europe followed the migrations of its *M. glareolus* host as already described for other hantavirus [104]. (1) PUUV ancestors probably originated from Asia, survived within lineages of bank voles during glaciation in several refugia, and spread independently with their respective hosts during postglacial recolonization. (2) different PUUV lineages could have also arise in a contact zones resulting from local reassortant and/or recombinant between PUUV lineages. The alternative scenario that a unique and common PUUV ancestor survived in only one refugium during glaciation, was later transmitted between distinct phylogenetic lineages of bank vole emerging from refugia during the post-glacial recolonization and micro-evolved with them may be attractive in line with the generally considered “star-like” phylogeny of PUUV. However, it is looking less probable since it would imply a much faster PUUV evolution rate.

## Supplementary Materials

The following are available online at https://www.mdpi.com/1999-4915/11/8/679/s1, Figure S1: Potential post-glaciation dispersal routes of PUUV in Europe, Table S1: Alternative phylogeographic scenarios of isolates from Baltic region, Russia, and North-Scandinavia inferred using the BEAST program, Table S2: Bayes Factors and Posteriors Probabilities supports for transitions rates between locations calculated by BEAST program during BSSVS procedure and summarized by SPREAD3 program.
