# Supplementary material for: Phylogeography of Puumala orthohantavirus in Europe
Source: Viruses. 2019 Jul 24;11(8):679. doi: 10.3390/v11080679 (PMC6723369; doi:10.3390/v11080679)
Supplement: Supplementary file 1 [file viruses-11-00679-s001.pdf]

Table S1. List of the coding sequences of the S segment used for phylogenetic analyses and ancestral reconstructions. Isolates sequenced for this study are indicated by a (*).

Strain Name	GenBank Accession Number	PUUV Lineage	Geographical Location
PUU/Ernstbrunn/Cg641/1995	AJ888752	ALAD	Alpe-Adria region
PUU/Klippitztoerl/Cg9/1995	AJ888751		
PUUV/Croatia_Gerovo/Mg938/2008	KC676609		
PUUV/Croatia_Gerovo/Mg955/2008	KC676611		
Balkan-1	AJ314600		
Balkan-2	AJ314601		
PUUV/Mg23/HungaryTR17/00	FN377822		
PUUV/Mg9/HungaryTR17/00	FN377821		
Opina916	AF294652	CE	
PUUV/NL/Mg1439/2011	KU314901		Netherland
PUUV/NL/Mg25/2007	KU314897		
PUUV/NL/Mg1012/2010	KU314900		
PUUV/NL/Mg753/2009	KU314899		
PUUV/NL/Mg31/2007	KU314896		
PUUV/NL/Mg343/2008	KU314898		
PUUV/NL/Mg591/2008	KU314905		
PUUV/NL/Mg2/2008	KU314902		
PUUV/NL/Mg502/2008	KU314904		
CG 13891	U22423		Belgium
CG14444	AJ277075		
Couvin/59Cg/97	AJ277034		
Momignies/47Cg/96	AJ277032		
Montbliart/23Cg/96	AJ277031		
Thuin/33Cg/96	AJ277030		
Bavaria 151/05	EU439968		Germany
Mu362Osnabrueck/05	JN696358		
MuEb10Karlstadt/10	JN696373		
MuEb12Lackenberg/10	JN696374		
MuEb51Elsenthal/10	JN696376		
Mu/07/1219	KJ994776		
PUU/Mignovillard/CgY02/2005	AM695638		France
PUUV/Ardennes/Mg156/2011	KT247592		
PUUV/Ardennes/Mg75/2011	KT247593		
PUUV/Jura/Mg2/2010	KT247596		
PUUV/Jura/Mg214/2010	KT247597		
PUUV/Orleans/Mg23/2010	KT247594		
PUUV/Orleans/Mg29/2010	KT247595		
Chaource/Troyes_2008_RP2	KY364996		
Murbach-Alsace_2015_17/96	KY365003		
Murbach-Alsace_2015_17/50	KY365002		
Murbach-Alsace_2015_16/94	KY365001		
Murbach-Alsace_2015_2/12	KY365000		
Septmoncel-Jura_2015_Camp5	KY365007		
Septmoncel-Jura_2015_Camp9	MK946425*		
Ardennes_2011_87	KY364995		
ChauxdesCrotenay-Jura_2014_CI11	KY364997		
ChauxdesCrotenay-Jura_2014_NCHA71	KY364998		
ChauxdesCrotenay-Jura_2014_DG12	MK946426*		
MontsousVaudrey-Jura_2014_NCHA14	KY364999		
Orleans_2014_NCHA373	KY365004		
Orleans_2014_NCHA376	MK946422*		
Orleans_2014_NCHA380	MK946423*		
Orleans_2012_ORW12_54	MK946428*		
Poligny-Jura_2015_B18	KY365005		

Poligny-Jura_2015_C05	KY365006		
Poligny-Jura_2014_NCHA181	MK946427*		
Poligny-Jura_2015_C17	MK946424*		
Vosges_LPP_2015_HA_L_3/25	MK946429*		
Ardennes_Hargnies_2011_136	MK946433*		
Ardennes_Hargnies_2011_161	MK946434*		
Ardennes_Hargnies_2011_5	MK946432*		
Ardennes_CSSP15-5_2015	MK946430*		
Ardennes_CSW15-31_2015	MK946431*		
Fyn	AJ238791		
Fyn47	AJ278092	DAN	Denmark
Fyn131	AJ278093		
Evo/12Cg/93	Z30702		
Evo/13Cg/93	Z30703		
Kuhmo/X11	GU808825		
Pallasjarvi/63Cg/98	AJ314597		
Puu/Puu/1324Cg/79	Z46942		
Puu/Virrat/25Cg/95)	Z69985		Finland
PUUV/Konnevesi/Mg_M114B/2005	JQ319171		
PUUV/Konnevesi/Mg_M94A/2005	JQ319163		
PUUV/Pieksamaki/human_kidney/2008	JN831950		
PUUV/Pieksamaki/human_lung/2008	JN831947	FIN	
PUUV/Pieksamaki/Mg7/2008	JN831943		
Sotkamo	NC005224		
Gomselga	AJ238790		
Karhumaki	AJ238788		
Kolodozero	AJ238789		
CG144	AF367064		
CG168	AF367065		
CRF161	AF367069		
CRF308	AF367070		
CRF366	AF367071		Russia
CG1820	M32750		
DTK/Ufa-97	AB297665		
Samara_49/CG/2005	AB433843		
Samara_94/CG/2005	AB433845		
Udmurtia/338Cg/92	Z30708		
Udmurtia/894Cg/91	Z21497		
Baltic/205Cg/00	AJ314599	RUS	
Baltic/49Cg/00	AJ314598		
PUUV/Jelgava/Mg136/2008	JN657230		
PUUV/Jelgava/Mg140/2008	JN657231		
PUUV/Madona/Mg233/2008	JN657232		
PUUV/Madona/Mg99/2008	JN657229		Baltic countries
LT15/174	KX757840		
LT15/164	KX757839		
LT15/201	KX757841	LAT	
PUUV/Jelgava/Mg149/2008	JN657228		
KS13/855	KX815394		Poland
KS14/118	KX815395		
Aijajarvi/Mg9/05	GQ339477		
Bussjo_95-1	AM746297		
Bussjo_98-1	AM746300		
Djaknebole_98-1	AM746310		
Djaknebole_98-2	AM746311		
Gumboda_98-1	AM746315	N-SCA	North-Scandinavia
Gumboda_98-2	AM746316		
Gyttjea/Mg19/05	GQ339480		
Jockfall/Mg12/05	GQ339478		
Kalvudden/Mg22/05	GQ339482		
Kiviniemi/Mg3/05	GQ339473		
Ljustrask/Mg20/05	GQ339481		

Moskosel/Mg17/05	GQ339479		
Norum_98-1	AM746320		
Norum_98-2	AM746321		
Palbole_98-1	AM746327		
Palbole_98-2	AM746328		
Puu/Huggberget/Cg36/94	AJ223371		
Puu/Mellansel/Cg47/94	AJ223374		
Puu/Mellansel/Cg49/94	AJ223375		
Puu/Tavelsjo/Cg81/94	AJ223380		
Puu/Vindeln/L20Cg/83	Z48586		
Skaran_98-1	AM746331		
Skaran_98-2	AM746332		
Umea/hu	AY526219		
Bergsjobo/Mg25/05	GQ339483	S-SCA	South-Scandinavia
Faboviken/Mg26/05	GQ339484		
Mangelbo/Mg1/05	GQ339485		
Munga/Mg16/05	GQ339487		
Munga/Mg2/05	GQ339486		
Puu/Eidsvoll/1124v	AJ223368		
Puu/Eidsvoll/Cg1138/87	AJ223369		
Puu/Solleftea/Cg3/95	AJ223376		
Puu/Solleftea/Cg6/95	AJ223377		
00-18	DQ138128	MUJV	Asia
11-1	JX028273		
11-4	JX046484		
11-5	JX046487		
Fusong843-06	EF488805	FUSV	
Fusong84-05	EF211819		
Fusong199-05	EF488803		
Fusong 200-05	EF211820		
Fusong114-05	EF488804		
Fusong900-06	EF488806		
Fusong-Cr-247	EF442087		
Fusong-Cr-275	EF442091		
Kamiiso-8Cr-95	AB010730	HOKV	
Tobetsu-60Cr-93	AB010731		

Table S2. Bayes Factors and Posteriors Probabilities supports for transitions rates between locations calculated by BEAST program during BSSVS procedure and summarized by SPREAD3 program. Transitions supported by a BF > 2 are highlighted in yellow and transitions supported by BF > 5 are highlighted in orange.

FROM	TO	BAYES FACTOR	POSTERIOR PROBABILITY
Alpe-Adria region	Asia	1,95	0,27
Alpe-Adria region	Baltic countries	1,21	0,19
Alpe-Adria region	Belgium	1,84	0,26
Alpe-Adria region	Denmark	2,01	0,28
Alpe-Adria region	Finland	0,33	0,06
Alpe-Adria region	France	1,66	0,24
Alpe-Adria region	Germany	2,53	0,33
Alpe-Adria region	Netherland	1,44	0,22
Alpe-Adria region	North-Scandinavia	1,67	0,24
Alpe-Adria region	Poland	0,3	0,05
Alpe-Adria region	Russia	1,22	0,19
Alpe-Adria region	South-Scandinavia	2,78	0,35
Asia	Baltic countries	0,88	0,15
Asia	Belgium	0,3	0,05
Asia	Denmark	1,64	0,24
Asia	Finland	0,27	0,05
Asia	France	0,26	0,05
Asia	Germany	0,33	0,06
Asia	Netherland	0,33	0,06
Asia	North-Scandinavia	1,74	0,25
Asia	Poland	0,29	0,05
Asia	Russia	0,84	0,14
Asia	South-Scandinavia	1,35	0,21
Baltic countries	Belgium	0,25	0,05
Baltic countries	Denmark	1,13	0,18
Baltic countries	Finland	0,88	0,15
Baltic countries	France	0,21	0,04
Baltic countries	Germany	0,3	0,05
Baltic countries	Netherland	0,28	0,05
Baltic countries	North-Scandinavia	0,88	0,15
Baltic countries	Poland	92,11	0,95
Baltic countries	Russia	30,67	0,86
Baltic countries	South-Scandinavia	1,31	0,2
Belgium	Denmark	0,37	0,07
Belgium	Finland	0,16	0,03
Belgium	France	109751,59	1
Belgium	Germany	2,7	0,34
Belgium	Netherland	20,02	0,8
Belgium	North-Scandinavia	0,3	0,06
Belgium	Poland	0,18	0,03
Belgium	Russia	0,26	0,05
Belgium	South-Scandinavia	0,39	0,07
Denmark	Finland	0,37	0,07
Denmark	France	0,29	0,05
Denmark	Germany	0,4	0,07
Denmark	Netherland	0,39	0,07
Denmark	North-Scandinavia	2,1	0,29
Denmark	Poland	0,38	0,07
Denmark	Russia	1,12	0,18
Denmark	South-Scandinavia	1,71	0,25
Finland	France	0,14	0,03
Finland	Germany	0,17	0,03
Finland	Netherland	0,17	0,03
Finland	North-Scandinavia	0,32	0,06
Finland	Poland	0,18	0,03
Finland	Russia	13714,45	1
Finland	South-Scandinavia	0,35	0,06
France	Germany	2,53	0,33
France	Netherland	1,52	0,23
France	North-Scandinavia	0,26	0,05
France	Poland	0,15	0,03
France	Russia	0,21	0,04
France	South-Scandinavia	0,31	0,06
Germany	Netherland	7,78	0,6
Germany	North-Scandinavia	0,35	0,06
Germany	Poland	0,19	0,04
Germany	Russia	0,27	0,05
Germany	South-Scandinavia	0,46	0,08
Netherland	North-Scandinavia	0,32	0,06
Netherland	Poland	0,19	0,03
Netherland	Russia	0,26	0,05
Netherland	South-Scandinavia	0,43	0,08
North-Scandinavia	Poland	0,31	0,06
North-Scandinavia	Russia	0,82	0,14
North-Scandinavia	South-Scandinavia	1,25	0,2
Poland	Russia	0,39	0,07
Poland	South-Scandinavia	0,35	0,06
Russia	South-Scandinavia	1,24	0,19

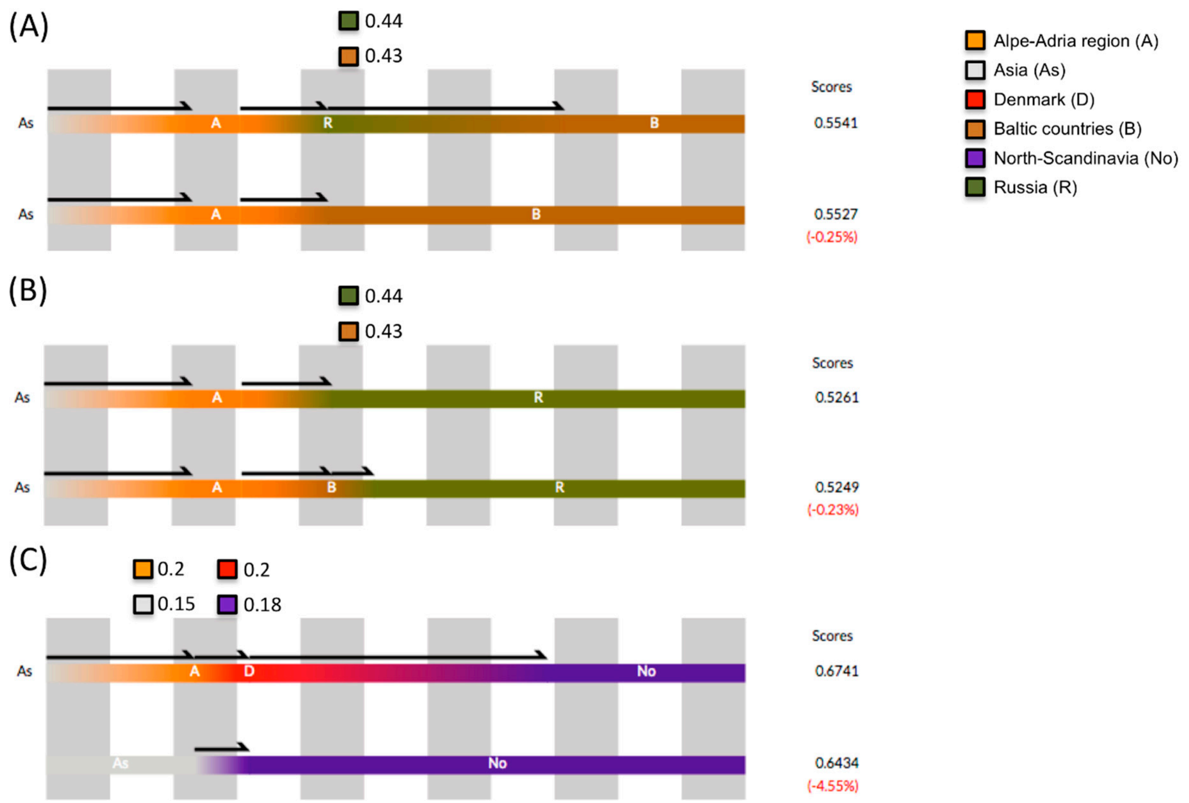

Figure S1. Alternative phylogeographic scenarios of isolates from Baltic region (A), Russia (B), and North-Scandinavia (C) inferred using the BEAST program. The different scenarios and their corresponding scores are visualized with AQUAPONY program [63]. The values associated to the colored squares represent the probability of the corresponding location for each scenario (only differences between the two scenario are thus shown). Black arrows represent the transitions between two locations.
